# Radiocesium concentrations in wild mushrooms after the accident at the Fukushima Daiichi Nuclear Power Station: Follow-up study in Kawauchi village

**DOI:** 10.1038/s41598-017-05963-0

**Published:** 2017-07-27

**Authors:** Makiko Orita, Kanami Nakashima, Yasuyuki Taira, Toshiki Fukuda, Yoshiko Fukushima, Takashi Kudo, Yuko Endo, Shunichi Yamashita, Noboru Takamura

**Affiliations:** 1 Department of Global Health, Medicine and Welfare, Atomic Bomb Disease Institutte, Nagasaki University, Nagasaki, 8528523 Japan; 2 Department of Radioisotope Medicine, Atomic Bomb Disease Institute, Nagasaki University, Nagasaki, 8528523 Japan; 3 Department of Radiation Medical Sciences, Atomic Bomb Disease Institute, Nagasaki University, Nagasaki, 8528523 Japan; 4 Department of Nursing, Nagasaki University Graduate School of Biomedical Sciences, Nagasaki, 8528523 Japan; 5 Kawauchi Municipal Government, Fukushima, 9791201 Japan

## Abstract

Since the accident at the Chernobyl Nuclear Power Plant, it has become well known that radiocesium tends to concentrate in wild mushrooms. During the recovery process after the accident at the Fukushima Daiichi Nuclear Power Station (FDNPS), it is important to perform follow-up measurements of the activity concentrations of radiocesium in mushrooms. We evaluated the activity concentrations of the detected artificial radionuclides (radiocesium) in wild mushrooms collected from Kawauchi village, which is within 30 km of the FDNPS, in 2015, four years after the accident. We found that the radiocesium was determined in 147 of 159 mushroom samples (92.4%). Based on the average mushroom consumption of Japanese citizens (6.28 kg per year), we calculated committed effective doses ranging from <0.001 to 0.6 mSv. Although committed effective doses are relatively limited, even if residents have consumed mushrooms several times, continuous monitoring of the radiocesium in mushrooms in Fukushima is needed for sustained recovery from the nuclear disaster.

## Introduction

The Great East Japan Earthquake occurred on 11 March 2011, and the resulting tsunami triggered a nuclear reactor accident at the Fukushima Daiichi Nuclear Power Plant Station (FDNPS). Due to this accident, huge amounts of radionuclides, including radioiodine and radiocesium, have been released into the environment. The United Nations Scientific Committee on the Effects of Atomic Radiation (UNSCEAR) estimated the total amount of released radionuclides to the atmosphere for iodine-131 (^131^I), cesium-134 (^134^Cs), and cesium-137 (^137^Cs) at 120.0, 9.0, and 8.8 petabecquerel (PBq), respectively^1^.

After the accident, to minimize internal exposure from consuming contaminated foods, the Japanese and prefectural governments initiated food monitoring, including of milk, vegetables, grains, meat, and fish, and other foods containing radioactive materials that exceeded the provisional regulation values were prohibited from distribution and consumption^1–5^. Due to these policies, internal exposure doses among the residents of Fukushima were relatively limited. Internal radiation exposure doses from the activity concentration of artificial radionuclides have been evaluated by whole body counters (WBCs) in Fukushima Prefecture^3–7^. Fukushima Prefecture reported the results of internal radiation doses measured from June 2011 to October 2016 in 307,208 individuals. Of these, 307,182 (99.9%) showed a committed effective dose less than 1 mSv. These results suggested that internal radiation exposure doses among the residents of Fukushima are limited thanks to food-monitoring policies.

**Figure 1:**
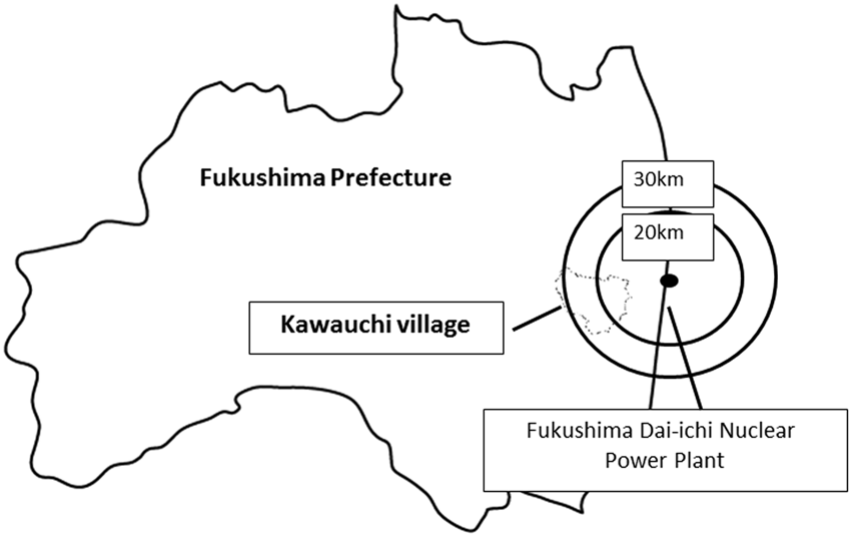
Location of Kawauchi village, Fukushima Prefecture, Japan.

On the other hand, it is well known since the accident at the Chernobyl Nuclear Power Plant that radiocesium tends to concentrate in wild mushrooms^8–14^. Recently, we evaluated the activity concentration of the detected artificial radionuclides (radiocesium) in wild mushrooms collected in Kawauchi village (Fig. 1), located within 30 km of the FDNPS, and we found radiocesium activity concentrations exceeding 100 Bq/kg (the current regulatory limit for radiocesium for general foods) in 125 of 154 mushrooms (81.2%) collected in 2013, two years after the accident^15^. During the recovery process following the accident at the FDNPS, it is important to perform follow-up measurements of the radiocesium in mushrooms, to monitor the dynamics of radiocesium in the environment, and to minimize the internal radiation exposure of residents of Fukushima through the consumption of contaminated foods. In this study, we evaluated the radiocesium in wild mushrooms in Kawauchi village collected in 2014 and 2015, three and four years after the accident.

## Results

**Figure 2:**
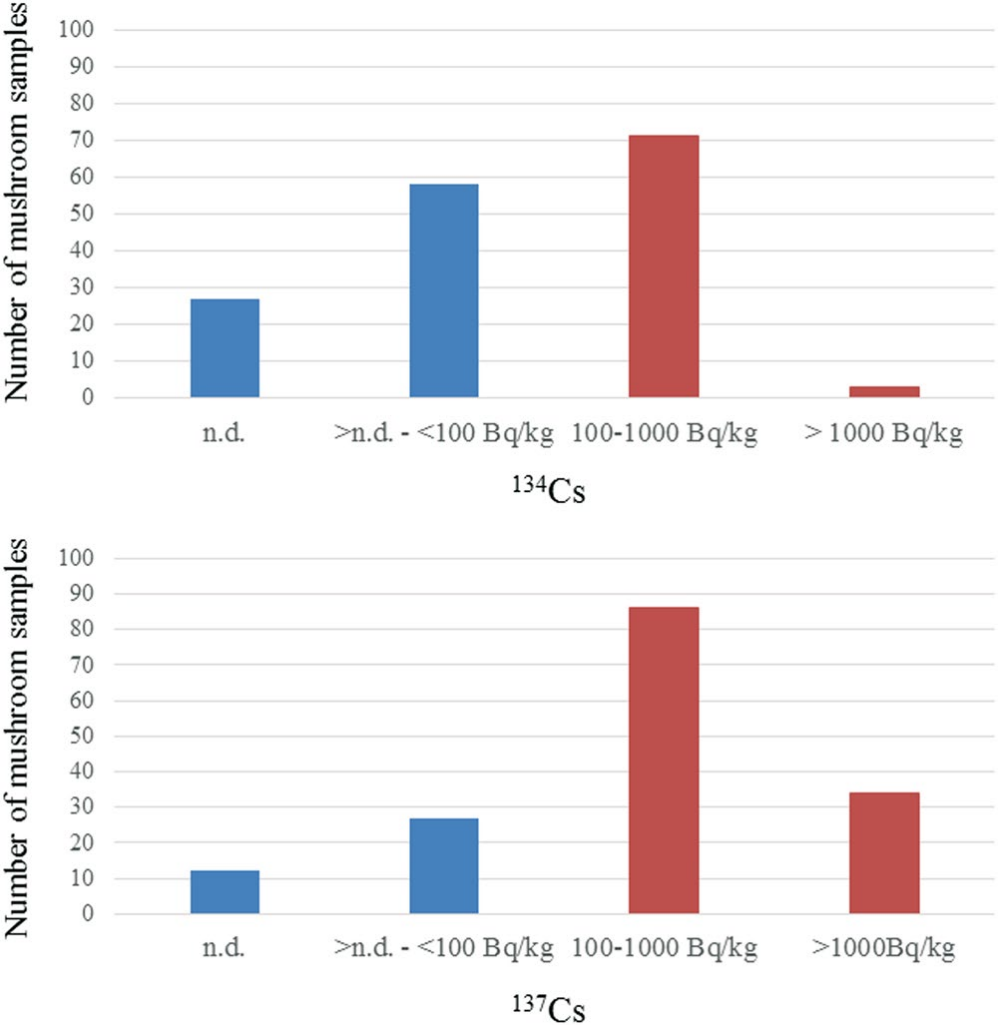
Distribution of concentrations of radiocesium in all samples collected in 2015.

The activity concentrations of radiocesium [the sum of individual activity concentrations (^134^Cs + ^137^Cs)] in mushrooms collected in 2015 are summarized in Fig. 2. The activity concentration of radiocesium was detected in 147 of 159 mushroom samples (92.5%). Among them, less than 99 Bq/kg of radiocesium was detected in 24 mushroom samples (15.1%), 100–999 Bq/kg was detected in 80 mushroom samples (50.3%), and more than 1,000 Bq/kg was detected in 43 mushroom samples (27.0%). On the other hand, radiocesium was not detected in 12 mushroom samples (7.5%).

The activity concentrations of radiocesium [the individual activities of ^134^Cs and ^137^Cs and the sum of individual activity concentrations (^134^Cs + ^137^Cs)] in each species of mushroom samples collected in 2015 are summarized in Table 1. Radiocesium concentrations was detected in 68 of 68 samples (100%) of *Sarcodon aspratus*, 17 of 17 samples (100%) of *Hygrophorus russula*, and 9 of 10 samples (90%) of *Albatrellus confluens*. The maximum activity concentration was 2,200 Bq/kg of ^134^Cs in *Hygrophorus russula* and 5,600 Bq/kg of ^137^Cs in *Sarcodon aspratus*. Radiocesium was not detected in *Lyophyllum decastes* (n = 4), *Pholota squarrosa* (n = 2), *Lyophyllum shimeji* (n = 1), and *Grifola frondosa* (n = 1)

**Table 1:** Concentrations of radiocesium in wild mushroom samples collected in 2015.

		n^*1^	^134^Cs Median (minimum–maximum) (Bq/kg)	Detection limit of ^134^Cs (median) (Bq/kg)	^137^Cs Median (minimum–maximum) (Bq/kg)	Detection limit of ^137^Cs (median) (Bq/kg)
*Symbiotic*	*Sarcodon aspratus*	68	150 (40–1,300)	10	620 (150–5,600)	8
	*Hygrophorus russula*	17	260 (40–2,200)	18	1,300 (20–3,900)	13
	*Albatrellus confluens*	10	n.e.^*2^ (<11–130)	11	40 (<8–550)	8
	*Lyophyllum fumosum*	7	n.e. (<10–110)	10	100 (<9–420)	9
	*Hypholoma sublateritium*	6	n.e. (<11–70)	11	100 (20–260)	9
	*Tricholoma equestre*	5	50 (30–60)	10	200 (150.0–300)	7
	*Boletopsis leucomelas*	5	80 (30–140)	13	450 (200–600)	9
	*Cortinarius salor Fr*.	4	270 (<21–600)	21	1,100 (30–2,400)	15
	*Cortinarius tenuipes*	4	n.d.^*3^ (<10–20)	10	45 (30–60)	7
	*Ramaria botrytis*	3	120 (40–140)	14	500 (200–730)	9
	*Entoloma sarcopum*	2	40 (20–60)	12	210 (120–300)	10
	*Suillus bovinus*	2	n.d.	9	n.d.	7
	*Leccinum extremiorientale*	2	60 (30–100)	11	350 (100–550)	9
	*Lyophyllum shimeji*	1	n.d.	11	n.d.	8
	*Clitocybe nebularis*	1	600	16	2,560.0	12
	*Lactarius hatsudake*	1	270	14	1,120.0	10
*Saprophytic*	*Armillaria mellea*	7	n.e. (<10–30)	10	70 (<7–820)	7
	*Lyophyllum decastes*	5	n.e. (<11–30)	11	n.e. (<10–140)	10
	*Armillaria tabescens*	3	20 (<8–50)	8	30 (<7–300)	7
	*Pholiota squarrosa*	2	n.d.	11	30 (30–40)	9
	*Pleurotus ostreatus*	2	10 (<10–20)	10	40 (20–60)	8
	*Lentinula edodes*	1	170	12	650.0	9
	*Grifola frondosa*	1	n.d.	13	n. d.	13

^*1^n: the number of mushroom samples. ^*2^n.e.: the median activity concentration of artificial radionuclides was not quantified. ^*3^n.d.: the activity concentration of artificial radionuclides could not be determined.

**Figure 3:**
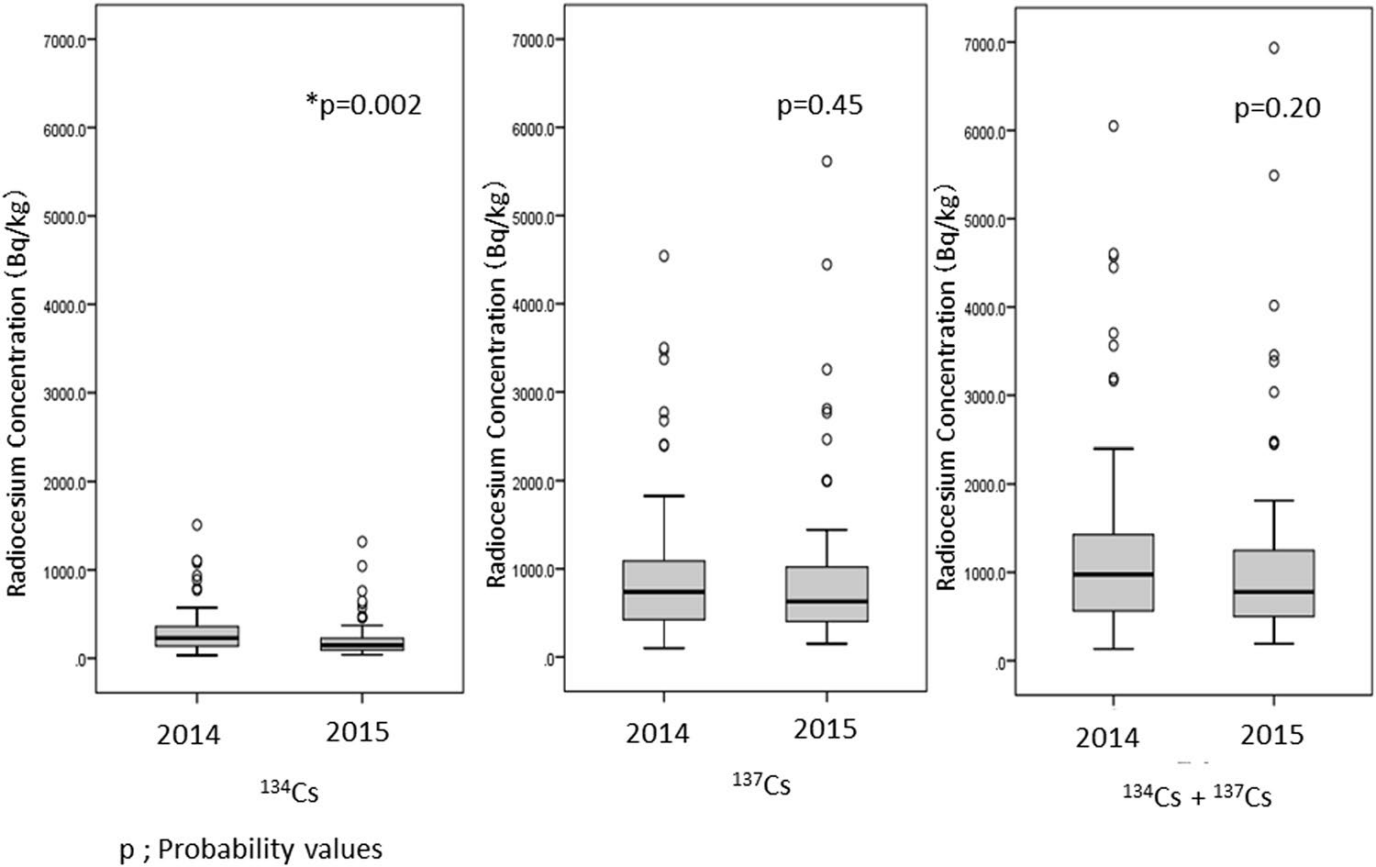
Comparison of concentrations of radiocesium of *Sarcodon aspratus* collected in 2014 and 2015.

In 2014, only 81 *Sarcodon aspratus* were collected, and in all of them activity concentration of radiocesium was detected. The maximum activity concentration of ^134^Cs in these samples was 1,500 Bq/kg, and the median and minimum activity concentrations were 230 and 30 Bq/kg, respectively; the maximum activity concentration of ^137^Cs was 4,500 Bq/kg, and the median and minimum activity concentrations were 740 and 100 Bq/kg, respectively. We compared the activity concentrations of radiocesium in the *Sarcodon aspratus* sampling collection between 2014 (n = 81) and 2015 (n = 68) to evaluate the trend of radiocesium concentrations in the same species (Fig. 3), the concentrations of ^134^Cs in 2015 were significantly lower than those in 2014 (p = 0.002), whereas there was no difference in the concentrations of ^137^Cs between 2014 and 2015 (p = 0.45).

**Figure 4:**
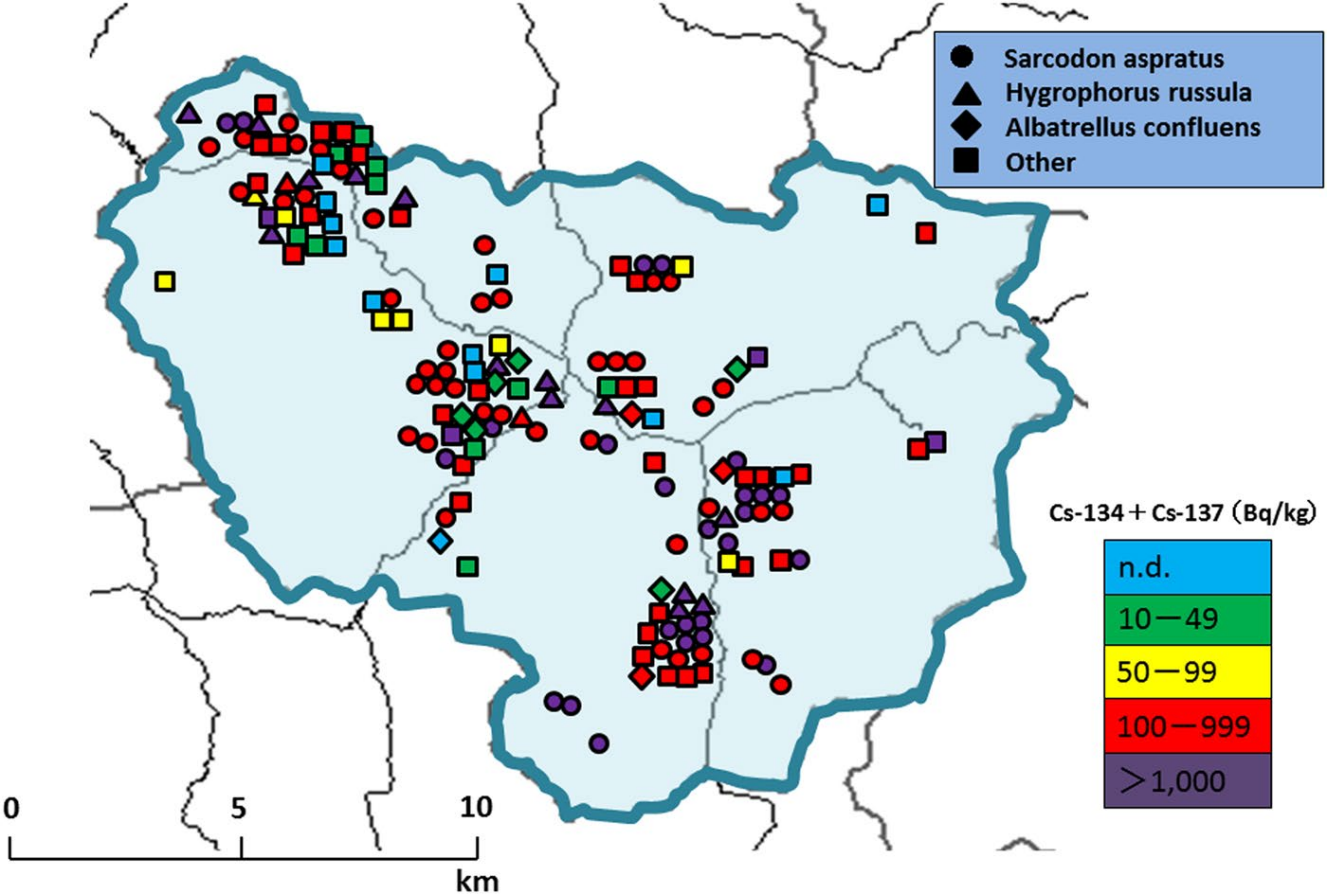
Sampling spots color coded by the concentration of radiocesium in mushrooms collected at each location in 2015. The bottom and top ends of the box and the bar inside the box correspond to the 25^th^, 75^th^, and 50^th^ sample percentiles, respectively. The circles with black dots represent extreme values.

Next, we mapped the distribution of mushrooms with radiocesium collected in 2015 (Fig. 4). No clear relationship was observed between sampling spots and cesium concentrations. Finally, we calculated committed effective doses, as shown in Table 2. Among the 147 mushroom samples collected in 2015 that contained detectable activity concentrations of radiocesium, we calculated committed effective doses ranging from <0.001 to 0.6 mSv.

**Table 2:** Committed effective dose due to wild mushroom intake in 2015.

	Species	Number of samples with detectable radiocesium	Committed effective dose, Median (minimum–maximum) mSv^*2^
*Symbiotic*	*Sarcodon aspratus*	68	0.07 (0.02–0.6)
	*Hygrophorus russula*	17	0.1 (0.006–0.4)
	*Albatrellus confluens*	10	0.004 (0.002–0.06)
	*Lyophyllum fumosum*	7	0.01 (0.001–0.05)
	*Hypholoma sublateritium*	6	0.01 (0.002–0.03)
	*Tricholoma equestre*	5	0.02 (0.02–0.03)
	*Boletopsis leucomelas*	5	0.05 (0.02–0.07)
	*Cortinarius salor Fr*.	4	0.1 (0.004–0.3)
	*Cortinarius tenuipes*	4	0.004 (0.004–0.007)
	*Ramaria botrytis*	3	0.06 (0.02–0.08)
	*Entoloma sarcopum*	2	0.02 (0.01–0.03)
	*Suillus bovinus*	2	0.004 (0.004–0.004)
	*Leccinum extremiorientale*	2	0.04 (0.02–0.06)
	*Lyophyllum shimeji*	0	Not evaluated
	*Clitocybe nebularis*	1	0.3
	*Lactarius hatsudake*	1	0.1
*Saprophytic*	*Armillaria mellea*	7	0.01 (0.003–0.07)
	*Lyophyllum decastes*	1	0.02
	*Armillaria tabescens*	2	0.02 (0.002–0.03)
	*Pholiota squarrosa*	0	Not evaluated
	*Pleurotus ostreatus*	2	0.005 (0.002–0.008)
	*Lentinula edodes* (*wood*)	1	0.07
	*Grifola frondosa*	0	Not evaluated

## Discussion

Recently, we evaluated the radiocesium in wild mushroom samples collected in Kawauchi village and found that radiocesium exceeding 100 Bq/kg was detected in 125 of 154 mushroom samples (81.2%) collected in 2013, two years after the FDNPS accident^15^. In our current study, radiocesium concentrations were detected in 123 of the 159 mushroom samples (77.4%) collected in 2015, four years after the accident. These results suggest that the portion of mushroom samples with radiocesium concentrations above 100 Bq/kg did not dramatically change in Fukushima over two years. In accordance with the shorter half-life of ^134^Cs, the activity concentrations of ^134^Cs (half-life = 2.1 years) in 2015 were significantly lower than those found in 2014, whereas there was no difference in the activity concentrations of ^137^Cs (half-life = 30.1 years) between 2014 and 2015. Although the residential houses have been extensively decontaminated since the accident, the forests of Fukushima Prefecture have not been decontaminated yet^15^. As our results suggest that it takes time to observe a decrease in the radiocesium in wild mushrooms, careful discussion will be needed among stakeholders to determine the necessity of decontaminating the forests in Fukushima.

On the other hand, we calculated committed effective doses ranging from <0.001 to 0.6 mSv based on the average annual intake of mushrooms by Japanese citizens. Previously, we calculated effective doses ranging from 0.1–1.60 mSv in 2013^15^. These results suggest that internal radiation exposure due to the consumption of wild mushrooms remained relatively limited in Kawauchi village. We have evaluated the activity concentrations of radiocesium in local foods produced in the village in 2013 and 2014. We showed that the number of samples exceeding the regulatory radiocesium limit (100 Bq/kg for general foods) was five of 4,080 sampled vegetables (0.1%), 652 of 1986 (32.8%) sampled edible wild plants and fungi, and eight of 647 (1.2%) sampled fruits. In addition, the internal radiation doses resulting from ingesting these foods ranged from 24.4 to 42.7 μSv for males and from 21.7 to 43.4 μSv for females^16^; this confirms that the internal radiation doses are acceptably low compared to the public dose limit of 1 mSv/year^16, 17^. Although residents who returned to the village may have a higher chance of consuming locally produced foods, it does not increase the meaningful of internal doses.

Kawauchi village is the first local authority to return to its hometown following the evacuation after the accident^18^. Currently, almost 68% of residents returned to the village and restarted their lives. Before the accident, the village was famous for its wild mushrooms, including *Sarcodon aspratus* and *Tricholoma matsutake*. Because the collection and consumption of wild mushrooms is a part of the culture of this village, residents are keenly interested in the radiocesium in the wild mushrooms. In 2013, we began collaborating with residents to prepare a “mushroom map” that includes information about radiocesium concentrations in the mushrooms collected in the village^11^. During the recovery phase from the nuclear disaster, the engagement of stakeholders, including residents, local authorities, and scientists, is important to deciding the future direction of the community. The International Commission on Radiation Protection (ICRP) emphasizes that stakeholder engagement is key to the development and implementation of radiological protection strategies for most existing exposure situations, and as experience in stakeholder engagement has grown, it has become possible to use many of the lessons learned as a basis for the development of best practices among the radiation protection community^19^. We believe our collaboration in Kawauchi village will contribute to the development of such practices.

Our study has several limitations. We could not evaluate the relationship between radiocesium activity concentrations in mushroom samples and the concentrations in soil due to insufficient soil samples. Further comprehensive studies are necessary to evaluate the activity concentrations of radiocesium in mushroom samples in Fukushima after the accident. Additional analytical uncertainties arise because the committed effective doses from dietary intake of mushrooms cannot measure day-to-day variations in individuals. Further, we did not evaluate the potential loss of radiocesium upon cooking in mushrooms. The influence of eating habits, including cooking methods, must be considered. In this study, we evaluated internal doses from the ingestion of mushrooms, but we did not evaluate the external doses received from being in contaminated forest areas while collecting mushrooms. Further comprehensive analyses with detailed reports on all areas around FDNPP are needed.

In conclusion, we evaluated the activity concentrations of radiocesium in wild mushrooms in Kawauchi village collected in 2014 and 2015, three and four years after the accident, and we confirmed that radiocesium was still detected in most samples. We explained our current results to the residents in the village. Although committed effective doses are relatively limited, we believe that continuous monitoring of the activity concentrations of radiocesium in mushrooms and risk communication with residents in Fukushima is needed for sustained recovery from the nuclear disaster.

## Materials and Methods

### Sampling of mushrooms

All wild mushrooms were collected in Kawauchi Village (the public office, N37° 20′, E140°48′), Fukushima Prefecture, Japan. Kawauchi village is located approximately 20 to 30 km southwest of the FDNPP and was partially included in the Evacuation Order Area (within a 20-km radius of the FDNPP). During the initial phase of the accident at the FDNPP, almost all residents were evacuated from the village. Saito *et al*. reported soil deposition density maps of gamma rays that were constructed on the basis of results from large-scale soil sampling in June 2011; these maps showed levels of artificial radionuclides of 100–300 kBq/m^2^ for ^134^Cs and 100–300 kBq/m^2^ for ^137^Cs in Kawauchi village^20^. On 31 January 2012, the head of Kawauchi village declared that residents who resided at least 20 km away from the FDNPP could return to their homes based on the declaration by the Japanese Prime Minister that the FDNPP reactors had achieved a state of “cold shutdown” in December 2011^18^. The Evacuation Order in its entirety (within a 20 km radius of the FDNPP) was lifted in June 2016, at which time all of residents in the village could return to their hometowns.

Mushrooms have been sampled every year during mushroom season (summer–autumn) since 2013. We asked residents of the village to collect mushrooms and to show the location of each mushroom. We collected 81 *Sarcodon aspratus* mushroom samples from September to November of 2014, and 159 mushroom samples of 23 species from September to November of 2015. Among the 159 mushroom samples collected in 2015, 68 (43.0%) were *Sarcodon aspratus*, 17 (10.7%) were *Hygrophorus russula*, and 10 (6.3%) were *Albatrellus confluens* (Table 1). The variety of collected mushrooms is quite wide, but some of these are not well known or easy to find, so they were not included in the sampling as they are less likely to be collected for ingestion. All mushroom samples collected were classified according to type into their typical categories of saprophytic or symbiotic, as shown in Table 1.

After collection, all samples were washed by water to remove the soil, and broken into smaller pieces using a mixer machine. The samples, approximately 41 g each wet weight (median), were enclosed in 100-mL plastic containers made of polypropylene (inner diameter, 50 mm; height, 14–42 mm) for radionuclide measurements. All samples were measured fresh and analyzed with a high-purity germanium detector (ORTEC^®^, GMX30–70, Ortec International Inc., Oak Ridge, TN, USA) coupled with a multi-channel analyzer (MCA7600, Seiko EG&G Co., Ltd., Chiba, Japan) for 3,600 s. The measuring time was set to detect the objective radionuclide, and the gamma-ray peaks used for the measurements were 604.66 keV for ^134^Cs (2.1 y) and 661.64 keV for ^137^Cs (30 y). Decay corrections were made based on the sampling date, and detector efficiency calibration was performed for different measurement geometries using mixed-activity standard volume sources (Japan Radioisotope Association, Tokyo, Japan). Activity concentrations of radiocesium were automatically adjusted based on the date of collection, and the data were defined as the activity concentrations at the collection date. The relative efficiency was 31%, and energy resolution of the spectrometer was 1.85 keV for ^60^Co. The detection limit was 11.5 Bq/kg for ^134^Cs and 9.2 Bq/kg for ^137^Cs (median), and counting errors were ±7.3 Bq/kg for ^134^Cs and ±14.1 Bq/kg for ^137^Cs, respectively. Sample collection, processing, and analysis were executed in accordance with standard methods of radioactivity measurement authorized by the Ministry of Education, Culture, Sports, Science, and Technology, Japan^21^. All measurements were performed at Nagasaki University (Nagasaki, Japan). The sum of ^134^Cs and ^137^Cs concentrations was indicated as “activity concentration of radiocesium” in order to aid comparison with the current regulatory limit for radiocesium (100 Bq/kg for general foods), which is determined by the central governments of Japan. In most samples, mushrooms contained ^134^Cs and ^137^Cs. However, in some samples, only ^137^Cs was detected, because ^134^Cs concentrations were below the detection limit. For such samples, concentrations of ^137^Cs were indicated as “concentrations of radiocesium.”

### Committed effective doses

The committed effective dose from ^137^Cs and ^134^Cs due to mushroom consumption was calculated using the following formula:

$${H}_{int}={\rm{C}}\cdot {D}_{int}\cdot {\rm{e}}$$

where *C* is the activity concentration of the detected artificial radionuclide (radiocesium) (Bq/kg), *D*_*int*_ is the dose conversion coefficient for adult intake (age 20 and older, 1.9 × 10^−5^ mSv/Bq for ^134^Cs and 1.3 × 10^−5^ mSv/Bq for ^137^Cs), and *e* is the estimated value of annual intake from the latest statistical data issued by the Ministry of Health, Labour, and Welfare, Japan in 2015^22, 23^. From this report, annual intakes of mushrooms were estimated at 6.28 kg/year in Japanese citizens, which is based on mean values of adults ( > 20 y). We assumed that annual intake could be attributed to each species.

### Statistical analysis

Values of activity concentrations of radiocesium are presented as median, minimum and maximum. The number of samples of each type of mushroom are listed in Table 1. Analysis of variance (ANOVA) was performed to compare radiocesium activity concentrations in mushroom samples collected in 2014 with radiocesium activity concentrations in mushrooms collected in 2015. Probability values less than 0.05 were considered statistically significant. All statistical analysis was performed using SPSS statistics 22.0 (SPSS Japan, Tokyo, Japan).
